# Nuclear DNA Content Variation in Different Life Cycle Stages of Sugar Kelp, *Saccharina latissima*

**DOI:** 10.1007/s10126-022-10137-9

**Published:** 2022-07-26

**Authors:** Franz Goecke, Amelia Gómez Garreta, Rafael Martín–Martín, Jordi Rull Lluch, Jorunn Skjermo, Åshild Ergon

**Affiliations:** 1 Department of Plant Sciences, Faculty of Biosciences, Norwegian University of Life Sciences, Ås, Norway; 2 Laboratori de Botànica, Facultat de Farmàcia I Ciències de L’Alimentació, Institut de Recerca de La Biodiversitat (IRBio) & Centre de Documentació de Biodiversitat Vegetal (CeDocBiV), Universitat de Barcelona, Barcelona, Spain; 3 Department of Fisheries and New Biomarine Industries, SINTEF Ocean, Trondheim, Norway

**Keywords:** Laminariales, Macroalgae, Seaweed, C-value, Ploidy

## Abstract

Ploidy variants can be utilized to increase yield, introduce sterility, and modify specific traits with an economic impact. Despite economic importance of *Saccharina* species, their nuclear DNA content in different cell types and life stages remain unclear. The present research was initiated to determine the nuclear DNA content and intraindividual variation at different life cycle stages of the Laminarialean kelp *Saccharina latissima*. Nuclear DNA content in embryonic and mature sporophytes, released and unreleased zoospores, female, and male gametophytes from Sør-Trøndelag county in Norway were estimated by image analysis using the DNA-localizing fluorochrome DAPI and chicken’s red blood cells as a standard. DNA content of a total of 6905 DAPI-stained nuclei was estimated. This is the first study of nuclear DNA content which covered the life cycle of kelp. The lowest level of DNA content (1C) was observed in zoospores with an average of 0.76 pg. Male and female single spore gametophyte cultures presented higher average DNA content, more than double that of zoospores, suggesting the presence of polyteny. Female gametophyte nuclei were slightly larger and more variable in size than those of male gametophytes. The DNA content observed in embryonic sporophytes and in meristoderm cells from older sporophytes (1.51 pg) was 2C as expected and in the range of previously published studies of sporophytes of *S. latissima*. Mature sporophytes showed intra-plant variation with DNA content values ranging from 2-16C. The main difference was between meristoderm cells (mostly 2C) and cortical and medullary cells (2-16C).

## Introduction

Kelp species, which are generally species in the order Laminariales (e.g., *Alaria* Greville, *Ecklonia* Hornemann, *Laminaria* J.V. Lamouroux, *Macrocystis* C. Agardh, *Nereocystis* Postels & Ruprecht, *Saccharina* Stackhouse, *Undaria* Suringar), form extensive kelp forests in temperate regions, representing some of the most productive marine ecosystems in the world (Bolton 2010; Phillips et al. 2011). Several kelp species are also important economic resources, used for extraction of hydrocolloids, e.g., alginate, or used for food or feed (Westermeier et al. 2010; Buschmann et al. 2017). One of the kelp genera in Europe and North America, *Saccharina latissima* (Linnaeus) C.E. Lane, C. Mayes, Druehl & G.W. Saunders, is one of the most economically and ecologically important kelp species (Monteiro et al. 2019). In Norway, *S. latissima* (or sugar kelp) is the most frequently farmed kelp, with a cultivation potential along the Norwegian coast estimated to be 150–200 tonnes per hectar and year (Broch et al. 2019).

**Fig. 1:**
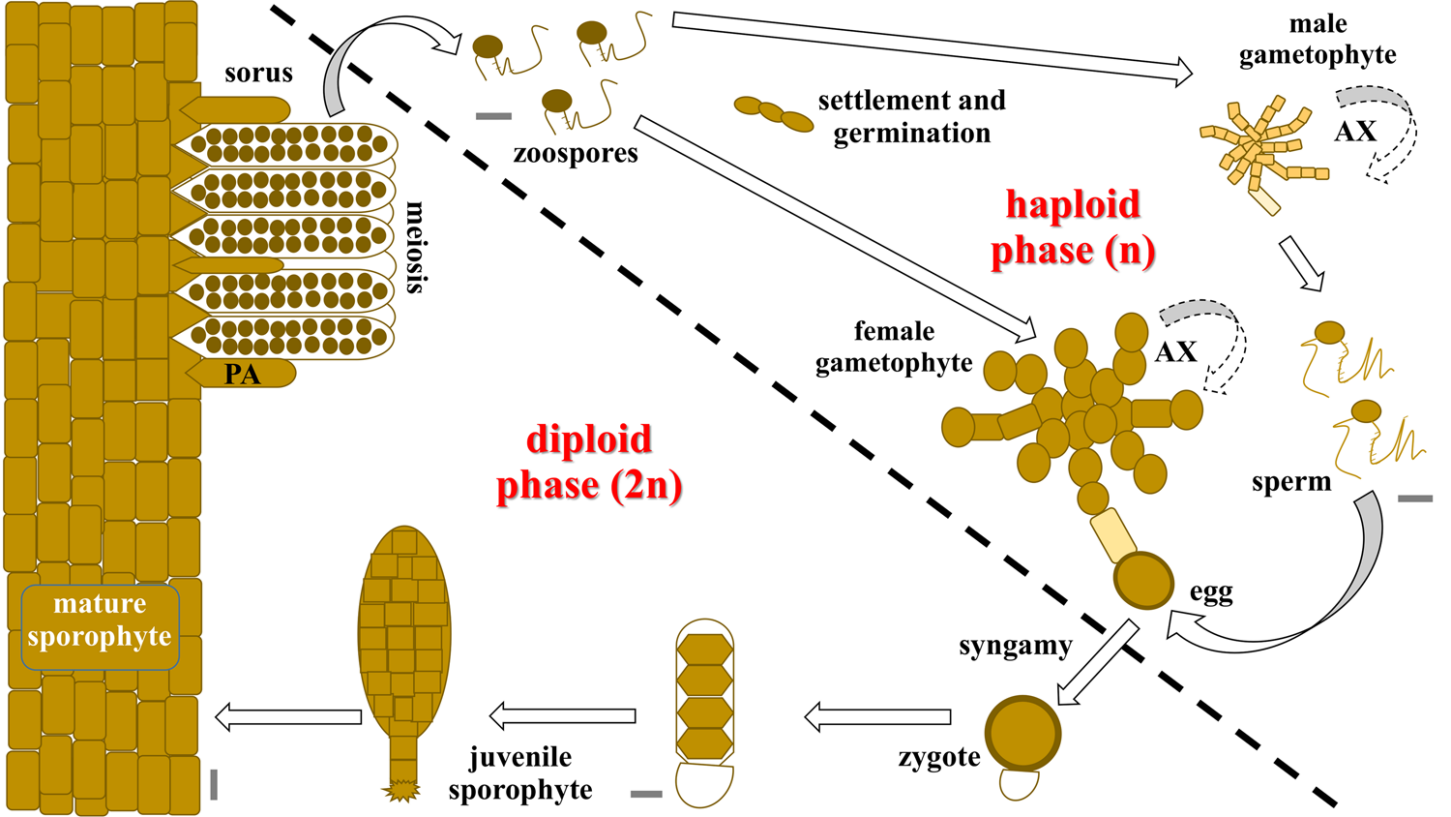
Typical biphasic life cycle of Laminarialean kelp. When mature, large diploid sporophytes develop sori over the lamina, composed of sporangia and paraphysis (PA). Motile zoospores, generated in the sporangia by meiosis, are further released into the water and develop into independent haploid female or male gametophytes. Both sexes can multiply vegetatively (AX) by disruption of the filaments. The production of eggs and sperms is followed by fertilization and the development of a new sporophyte. Scale bar 5 µm, although a large sporophyte can grow over 2 m long

In the marine environments, kelps represent a conspicuous group of macroalgae and there is an extensive body of literature focusing on their morphology, taxonomy, and ecology (Dayton 1985; Bolton 2010). The basic life cycle of species in the order Laminariales is biphasic, with an obligate cycling between separate and free-living haploid gametophytes and diploid sporophytes (Oppliger et al. 2014). Adult sporophytes produce haploid motile zoospores. Those zoospores settle and develop into free-living, dioecious microscopic gametophytes (in general, 50% male and 50% female) that produce oogamous, haploid gametes (eggs and sperm). Eggs are extruded from oogonia of female gametophytes, remain attached, and release pheromones that attract motile sperms from male gametophytes for fertilization. The zygote grows from the female gametophyte, attaches to a substrate, and develops into a multicellular sporophyte (Fig. 1). Additionally, a variety of asexual reproduction mechanisms have been reported for Laminarialean kelp species (see Goecke et al. 2020). Kelp sporophytes consist of a holdfast, stipe, and blade, each composed of different tissues (meristoderm, cortex and medulla; although specialized according to the specific area of the thallus) and may reach up to several meters in height (Monteiro et al. 2019). Classic anatomical studies from Sykes (1908), Drew (1910), Killian (1911), Naylor (1956), and Chung et al. (1987), described the internal structure of the sporophyte thallus of Laminarialean kelps like *S. latissima*. These authors described an outermost layer of small cuboidal, heavily pigmented cells (meristoderm). Below it, a cortical layer showing a gradual transition from almost isodiametric cells on the outside, to much more elongated ones on the inside, usually unpigmented and with large vacuoles. The medullary layer in the center of the thallus has a looser organization with entangled, very elongated, thread-like cells embedded in a mucilage. Furthermore, Kanda (1936) described the development in several Laminarialean kelps, including *Saccharina japonica* (Areschoug) C.E. Lane, C. Mayes, Druehl & G.W. Saunders. The author showed that size and shape of the gametophytes are very irregular, with smaller male gametophyte cells than the female gametophyte cells. In both cases, well-nourished gametophyte cultures grow into rather profusely branched filaments. There are several studies concerning the detailed ultrastructure of zoospores, gametophytes, gametes, and sporophytes from Laminariales (see Davies et al. 1973; Henry and Cole 1982a, b; Motomura and Sakai 1984; Holzinger et al. 2011). While we know the histological structure of kelp, we know little about the variation in nuclear DNA content among different tissue types and life stages.

Other studies used the number of chromosomes to confirm the ploidy level of gametophytes and sporophytes in different *Saccharina* species (*S. japonica*, *S. japonica* var. *diabolica* (Miyabe) N. Yotsukura, S. Kawashima, T. Kawai, T. Abe & L.D. Druehl, *S. latissima*, *S. longissima* (Miyabe) C.E. Lane, C. Mayes, Druehl & G.W. Saunders, and *S. yendoana* (Miyabe) C.E. Lane, C. Mayes, Druehl & G.W. Saunders). In general, there are 31 chromosomes in haploid gametophytes and 64 chromosomes in diploid sporophytes, although the number can vary slightly among species (Evans 1965; Cole 1967, 1968; Liu et al. 2012). DNA-content can also be used to infer the ploidy level. A C-value of 1 represents the amount of nuclear DNA in the non-replicated haploid chromosome complement (Greilhuber et al. 2005). Hence, a nucleus in G1-phase of the cell cycle, with two copies of unreplicated genome has a DNA amount of 2C (Doležel and Bartoš 2005). The nuclear DNA content is used in a wide range of biological fields, e.g., as a predictor of phenotypic characters at cell, tissue, or organism level (Bennett and Bhandol 2000). It also provides key information for a better understanding of the life history, and it has been related to patterns of adaptation, invasiveness, or evolution (Goff and Coleman 1990; Kapraun and Dunwoody 2002; Gómez Garreta et al. 2010; Ribera Siguan et al. 2011; Pellicer et al. 2018; Ĉertnerová and Škaloud 2020). Furthermore, eukaryotic genome size data are becoming increasingly important as direct estimators of the cost and difficulty of genome sequencing programs (Gregory et al. 2007).

Raven et al. (2019) recently reviewed the large range (by over four orders of magnitude) of the 1C value within higher taxa of algae (including micro- and macroalgae). The authors discussed how nuclear DNA content influences growth rate, the potential consequences of variation in cell size, mechanistic implications, and biophysical constraints imposed on the organisms. There is less published data on nuclear DNA contents, nuclear GC content, and genome complexity in macroalgae than in land plants (Le Gall et al. 1993; Salvador Soler et al. 2019). Kapraun (2005) presented an extensive overview on macroalgae C-values, including 111, 85, and 44 species and varieties of red, green, and brown algae, respectively. Later, Phillips et al. (2011) studied 98 species of brown algae of different phylogenetic orders. There have been other studies on C-values of brown macroalgae which include the phaeophycean orders Ectocarpales (Müller and Schmidt 1988; Deshmukhe and Tatewaki 1993; Le Gall et al. 1993; Peters et al. 2004), Dictyotales (Ribera Siguan et al. 2011), Fucales (Peters et al. 2004; Gómez Garreta et al. 2010; Sjøtun et al. 2017; Salvador Soler et al. 2019), and Sphacelariales (Le Gall et al. 1993). Salvador Soler et al. (2019) highlighted the low number of publications on DNA content of ecologically and economically important kelp species and Fucales. For the order Laminariales, which contains most of the economically important brown macroalgae, the C-value of only 12 species has been reported until now (Table 1); a small number of species, considering that Laminariales has, according to the database AlgaeBase, 137 taxonomically accepted species until now (Guiry and Guiry 2022). Not only is the number of studies (of DNA content) for this group of macroalgae limited, they are usually based on only a single life cycle stage.

**Table 1:** Nuclear DNA content with corresponding C-value in kelp species including Laminarialean species and one Fucalean species (*). Cell type correspond to gametophyte (Gp), female gametophyte (Gpf), male gametophyte (Gpm), sporophyte (Sp), cortical cell (Cc), sporangia (Sg), paraphysis (Pa), meristoderm (Me). Values are expressed in picograms (pg). If originally expressed in megabasepairs (Mb), we used the currently accepted conversion factor 1 pg = 980 Mb (Cavalier-Smith 1985)

Kelp species	Cell type	DNA amount			Reference
		**1C (pg)**	**2C (pg)**	**4C (pg)**	
*Agarum clathratum*	Sp	0.60	1.20	2.50	Kapraun (2005)
*Alaria esculenta*	Sp	0.70	1.20	2.30	Kapraun (2005)
Durvillaea incurvata*	Gpm	0.6	1.2	-	Salvador Soler et al. (2019)
D. incurvata* (as *D. antarctica*)	Sp-Pa, Cc	-	1.2	-	Salvador Soler et al. (2019)
*Ecklonia radiata*	Sp	0.60	1.30	2.60	Kapraun (2005)
*Egreria menziesii*	Sp	0.78	1.55	3.10	Phillips et al. 2011
*Laminaria digitata*	Gp/Sp	(0.64)-0.70	1.40	2.70–2.80	Le Gall et al. (1993), Marie and Brown (1993), Kapraun (2005)
*Laminaria hyperborea*	Sp	0.33	0.65	1.30	Phillips et al. (2011)
*Lessonia spicata*	Sp-Sg	0.8	-	-	Salvador Soler et al. (2019)
*L. spicata*	Sp-Me	0.9	1.5	-	Salvador Soler et al. (2019)
*Macrocystis pyrifera*	Gpm	1.05	2.10	4.20	Phillips et al. (2011)
*M. pyrifera*	Sp-Sg	0.7	-	-	Salvador Soler et al. (2019)
*M. pyrifera*	Sp-Cc	-	1.4	+	Salvador Soler et al. (2019)
*M. pyrifera* (as *M. angustifolia*)	Gpf	1.10	2.20	4.40	Phillips et al. (2011)
*M. pyrifera* (as *M. integrifolia*)	Sp	0.79	1.57	3.14	Phillips et al. (2011)
*Nereocystis luetkeana*	Sp	0.65	1.30	2.60	Phillips et al. (2011)
*Saccharina japonica*	Sp	0.59	-	-	Liu et al. (2019) (genomic analysis)
*S. latissima*	Gp/Sp	0.60 (0.72)–0.79	1.30 1.58	2.60 3.16	Kapraun (2005) Le Gall et al. (1993), Marie and Brown (1993)
*Undaria pinnatifida*	Gp/Sp	(0.58)–0.64 0.60	1.28 1.30	2.56 2.6	Le Gall et al. (1993), Marie and Brown (1993), and Kapraun (2005)
*U. pinnatifida*	Gpm	(0.52)	-	-	Shan et al. 2020 (genomic analysis)

The estimated 1C-value of species in the Phaeophyceae ranges from 0.102 to 1.837 pg (Le Gall et al. 1993; Kapraun 2005; Gómez Garreta et al. 2010; Phillips et al. 2011; Ribera Siguan et al. 2011). The range of 1C-value estimates for Ectocarpales is among the smallest, and those of Sphacelariales, Fucales, and Laminariales are among the largest (Kapraun 2005; Phillips et al. 2011). The *S. latissima* 1C-value is estimated, based on non-genomic methods, to be around 0.60–0.80 pg (Table 1). Very few genomic studies have been published for brown macroalgae so far; seven sequenced brown algae species have estimated 1C-values of 0.218 pg (Cock et al. 2010), 0.247 pg (Dittami et al. 2020), 0.556–0.592 pg (Ye et al. 2015; Liu et al. 2019), 0.40 pg (Wang et al. 2020), 0.157 pg (Nishitsuji et al. 2019), 0.52 pg (Shan et al. 2020), and 0.133–0.146 pg (Nishitsuji et al. 2016; 2020), in *Ectocarpus* sp., *Ectocarpus subulatus* Bft15b, *S. japonica*, *Sargassum fusiforme*, *Nemacystus decipiens*, *Undaria pinnatifida*, and four strains of *Cladosiphon okamurensis*, respectively.

Different studies of Laminarialean kelps have investigated the amount of nuclear DNA in cells at different life cycle stages, with contrasting results. Garman et al. (1994), using DAPI spectrofluorometry, found a large range of nuclear DNA levels (4C to 16C) in sporelings and gametophytes (with no sex distinction) of *Macrocystis pyrifera* (Linnaeus) C. Agardh. The authors showed that zoospores had four times the DNA level of sperms (4C vs. 1C), while gametophytes were 8C to 16C. Le Gall et al. (1993) and Ar Gall et al. (1996), using flow cytometry, found nuclear DNA levels ranging from 2 to 8C in sporophyte cells of *M. pyrifera*, *Laminaria digitata* (Hudson) J.V. Lamouroux, and *S. latissima*. The authors attributed level 1C to gametophytes with no sex distinction (Table 1). Garbary and Clarke (2002), using a DAPI spectrofluorimetric method, found 2C to 16C in sporophyte cells of *Alaria esculenta* (Linnaeus) Greville and *S. latissima*. 1C was attributed to zoospores and few-celled gametophytes of *A. esculenta*, the latter without sex distinction. It was not until Müller et al. (2016) that someone investigated the difference in nuclear levels (using flow cytometry) between female and male gametophytes cells of *M. pyrifera*. The authors found that nuclei of all female gametophytes contained approximately double the DNA content (2C) than that of males (1C) and suggested that this was due to polyteny in the females. Recently, Salvador Soler et al. (2019), using DAPI microfluorometric analysis, found nuclear DNA levels of 2C to 8C and 2C to 4C, in sporophyte cells of *M. pyrifera* and *Lessonia spicata* (Suhr) Santelices in González et al., respectively. The authors attributed level 1C to sporangial cells (unreleased zoospores, Table 1).

In the Phaeophyceae, changes in ploidy and transition between stages of the life cycle are not always coupled, and the “classic” idea that gametophytes and sporophytes are haploid and diploid, respectively, is not strictly applicable to these organisms (Bogaert et al. 2013). For example, Oppliger et al. (2014), using a combination of flow cytometry, epifluorescence, and microsatellite analyses, demonstrated that sporophytes from marginal populations of *L. digitata* showed a high propensity for producing unreduced (2n) zoospores, which further formed phenotypically normal male and female gametophytes with nuclear size consistent with 2n (2C) DNA contents. The authors suggested that automixis could be involved. Klimova and Klochkova (2021) recently found, by counting chromosomes, that representatives of *A. esculenta* at the Asian coast of the Pacific Ocean have a chromosome number which is half that of representatives of the Atlantic Ocean. The authors discussed the possibility that this feature can reflect the evolutionary origin of the species.

According to Kapraun (2005), larger nuclear DNA contents (≥ 2.0 pg) reported in the Fucales and Laminariales almost certainly represent polyploidy. There are several evolutionary mechanisms responsible for variation of the amount of nuclear DNA (Ĉertnerová and Škaloud 2020). As explained in Sjøtun et al. (2017) and Raven et al. (2019), a range of ploidy levels can be the result of rapid doubling of the genome from one generation to the next, in combination with further crosses or DNA reduction over time through various processes. Endoreduplication, for example, a modified cell cycle in which nuclear DNA is replicated without concomitant cell division, may be a key component of growth and development in many sessile organisms, including brown algae (Garbary and Clarke 2002; Bothwell et al. 2010). Several studies have reported natural polyploidy in macroalgae in general, and it has been widely reported for Phaeophyceae (Lewis 1996; Phillips et al. 2011), especially in the Laminariales (Lewis et al. 1993; Garbary and Clarke 2002), Ectocarpales (Deshmukhe and Tatewaki 1993; Bothwell et al. 2010), Fucales (Gómez Garreta et al. 2010), and Dictyotales (Ribera Siguan et al. 2011). Polyploidy has been related to environmental stress, asexual reproduction (Bothwell et al. 2010; Oppliger et al. 2014).

C-value differences have not only been observed between life cycle stages (e.g., gametophyte vs. sporophyte), between different reproduction processes (e.g., parthenogenesis vs. sexual reproduction), or between populations or individuals, but also internally in organisms. It has been shown that there is a close correlation between nuclear DNA content and cell volume in many lineages of eukaryotes, including animals, plants, and microalgae (Connolly et al. 2008). Specifically, it has been observed that the nuclear DNA content is proportional to the nuclear volume and that the nuclear volume is proportional to the cell volume (Jovtchev et al. 2006). Since there is a wide range of cell sizes in individual thalli of many brown algae, it is also possible that C-value variants are present, but this has been much less investigated in brown than in red algae (see Goff and Coleman 1990). In the case of multicellular algae, large cells are often polyploid through endoreduplication (Raven et al. 2019). This would explain the intra-thallus nuclear DNA content variation that has been documented previously in vegetative cells of Laminariales (Ar Gall et al. 1996; Garbary and Clarke 2002) and Fucales (Gómez Garreta et al. 2010).

Polyploidy has multiple applications in plant breeding, where inducing changes in ploidy is used as a tool to increase yield, modify other traits (vigor, adaptation, size, tolerance to stress or pathogens), or to introduce sterility and seedless fruits (Sattler et al. 2016). Understanding the natural variation in ploidy levels and the mechanisms behind this variation could be useful for developing tools that can be used in kelp breeding projects (Goecke et al. 2020). However, the future use of polyploids requires fundamental knowledge about the natural variation in ploidy levels and nuclear DNA content in these species. Due to reports of sex-related differences between gametophytes and difference between different tissues in adult sporophytes, the main objective of the present paper was to characterize the nuclear DNA content in the different life cycle stages of the commercially important kelp species *S. latissima*.

## Material and Methods

### Algal Material

Kelp sporophytes were collected by snorkeling at the subtidal zone around the island of Frøya in Sør-Trøndelag county (Mid-Norway), Norway (63°42′N, 08°51′E), in autumn 2018 and 2019, and in spring 2020. Heavily epiphyted blades were avoided. Kelp blades were transported in coolers at a temperature near 10 °C. For comparisons, one sample was also collected from each of two other distant locations in Norway in the summer of 2020: at Fedje (Nordhordland region of Vestland county; 60°46′N, 04°42′E) and Langesund (Vestfold and Telemark county, 58°59′N, 09°44′E). These two localities corresponded to two other ecoregions in Norway (Gundersen et al. 2017). Non-fertile fragments (and one fertile) were immediately preserved in methacarn fixative (3:1 of 95% methanol-glacial acetic acid) overnight to avoid staining inhibition by phenolic compounds and stored in 70% ethanol at 4 °C (Kapraun 2005).

Sporophyte samples from Frøya were used for sorus induction as described by Forbord et al. (2018). At arrival at the laboratory, sporophytes were cut 10–15 cm above the meristem and the distal end and sides were cut off. Sporophytes were washed in seawater to remove loosely attached epiphytes and maintained in 100-L circular flow-through tanks with 10 °C sand-filtered water, aeration and 8-h light from fluorescent, white light lamps. The sporophytes were inspected for sorus formation weekly. Fertile fragments were cut for disinfection of the sori and later we induced zoospore release as described previously (Bartsch 2018; Forbord et al. 2018). The zoospore solutions were immediately fixed with 2% formaldehyde in phosphate-buffered saline (PBS), and later washed with 100% PBS. After centrifugation of the zoospore solution (1000 rpm, 15 min), the precipitate was treated with methacarn fixative overnight, and stored in 70% ethanol at 4 °C.

Gametophyte cultures were obtained from fresh released zoospores (see above), placed into Falcon tubes containing glass microscope slides in seawater with 0.1 mL germanium dioxide (GeO_2_) L^−1^ for contamination control. Cultures were maintained at 12 °C using a light-tight climate room. Two-week-old filaments were handpicked under a stereomicroscope, sex-determined, and filaments were grown independently in MTP-24 well microplates. Gametophyte cultures were later upscaled into culture bottles in autoclaved artificial seawater supplemented with half-strength Provasoli solution (1/2PES SW, see West and McBride 1999). The culture flasks were left in constant red light with a light intensity of 20 µmol photons m^−2^ s^−1^ at the surface of the culture vessel (Bartsch 2018; Forbord et al. 2018). A few months later, grown gametophytes colonies were checked under the microscope, and healthy cultures were harvested and treated with methacarn fixative overnight, and stored in 70% ethanol at 4 °C.

We transferred fresh male and female clonal gametophyte strains to be crossed in Petri dishes under white light at around 30–60 µmol m^−2^ s^−1^ light intensity, with a 16:8-h light:dark regime and at an ambient temperature of 10 °C (Bartsch 2018; Forbord et al. 2018). Later, the algae were transferred under the same environmental conditions into 1-L glass bottles with Provasoli medium at constant aeration under filtered air flow, and at an initial pH of around 6.0. Sporophytes in early-stage development (embryonic, few-celled thalli up to maximum 1–2 mm long) was treated with methacarn fixative.

### Optical Microscopy

Light microscopy observations for all type of samples were performed using a Leica DM5000B microscope (Leica CTR5000, Leica Microsystems Limited, Heerbrugg, Switzerland) equipped with the attached Leica camera (DC200), and microphotographs were processed with the Leica Application Suite v4.3 image program (LAS 4.3).

### Cross sections of Sporophytes

Fresh sporophytes were mounted in a rotary microtome cryostat Leica CM1950 (Leica Biosystems Nussloch, Germany) set at a temperature of − 20 °C. Cryosections of 15 and 20 µm thickness were prepared using the cryostat microtome. The samples are embedded in Tissue Tek and frozen before section. The cryosections were mounted in microscope slides at room temperature and allowed to air dry.

### Transmission Electron Microscopy (TEM)

Samples from gametophytes were fixed for transmission electron microscopy using chemical fixation protocols according to Olsen et al. (2015). Briefly, filaments were harvested and prefixed in 1.25% glutaraldehyde and 2% formaldehyde in PBS solution for a minimum of 24 h. After washing several times in PBS and cacodylate buffer (0.1 M, pH 7.2), the cells were post-fixed for 1 h at room temperature in 1% osmium tetroxide (OsO_4_) and washed again in cacodylate buffer. After dehydration through an ethanol series (15 min in 70%, 15 min in 90%, 15 min in 96%, and 4 × 15 min in 100% ethanol), cells were infiltrated and embedded in in LR-White resin (Electron Microscopy Sciences, USA). Ultrathin sections were prepared using a Leica Ultramicrotome EM UC7, counterstained with 4% aqueous uranyl acetate and 1% potassium permanganate (KMnO_4_) for 5 min and then washed in distilled water before being examined and photographed in the transmission electron microscope (FEI Morgagni 268), using a Veleta CCD camera.

### Estimates of Nuclear DNA Content and Nucleus Relative Size

Estimates of nuclear contents were obtained from seven adult sporophytes, approximately 25 very young sporophytes (30 µm–2 mm), 11 gametophyte cultures (five female and six male cultures), unreleased zoospores from sporangia (1 parent), and three freshly released spore solutions (each from different parental individuals). Samples (treated with methacarn) were washed and rehydrated in distilled water and softened in 5% w/v EDTA for 1–72 h (Goff and Coleman 1990). Tissue samples were squashed and, like the spore solutions, transferred to coverslips treated with subbing solution, air dried, then briefly hydrated with 200 mM KCl, and stained with 0.5 µg mL DAPI (4′-6-diamidino-2-phenylindole; Sigma Chemical Co., St. Louis, Missouri, USA). Nuclear DNA contents were measured using fluorescence microscopy and image analysis, following a procedure modified from Kapraun and Dunwoody (2002) and Choi et al. (1994) (Salvador-Soler et al. 2019). The images obtained were then analyzed using Fiji software (US National Institutes of Health, Bethesda, Maryland, USA). The nuclear DNA content was obtained by comparison of the fluorescence intensity of the nuclei with that of a standard with constant nuclear DNA amount. Following Kapraun and Nguyen (1994) and Kapraun and Dunwoody (2002), we used *Gallus gallus* (Linnaeus) erythrocytes (from here on denoted as red blood cells, RBC) with constant nuclear DNA content of 2.4 picograms (pg) as standard (Kapraun 2005; Martín–Martín et al. 2020). SigmaPlot (version 14; Systat Software, Inc., San Jose, CA, USA) was used to plot the data and designate frequency distribution classes for further calculation of respective mean and standard deviations. For calculation of mean values of the nuclear DNA content, the peak frequency class was identified, and the neighboring frequency classes which presented frequencies above 50% of the peak frequency class were included.

A total of 6905 nuclei were localized and measured for macroalgae. Nuclear DNA content estimates were reported as C-value units, with 1C representing the estimated weight (pg ± SD of the total samples) of the nuclear DNA content of non-replicated zoospores obtained for *S. latissima* in Norway.

We measured relative nucleus size (100–380 nuclei each) and correlated this to nuclear DNA content at the different life cycle stages and tissue types (in mature sporophytes) using the mentioned image analysis Fiji software. This value corresponded to a relative value (without units), as we did not use a standard for nucleus (real) size but then again allowed us to correlate them with the DNA content obtained for each same nucleus of *S. latissima*.

Nuclear DNA content data obtained herein will be incorporated into the database of plant genome sizes compiled and hosted by the Royal Botanic Gardens (RBG) Kew web page (Leitch et al. 2019).

## Results

**Fig. 2:**
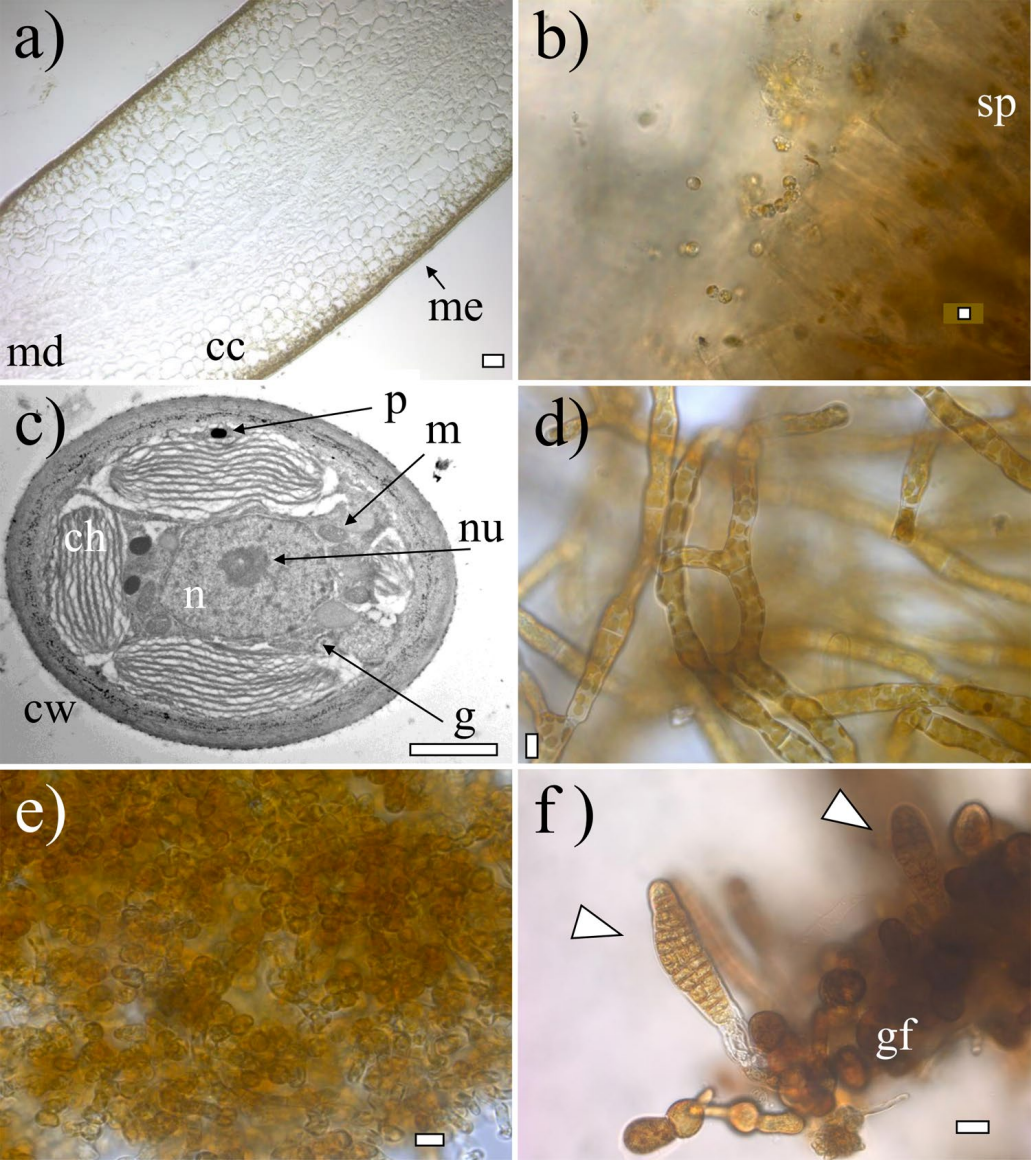
Morphology and anatomy of *Saccharina latissima*. **a** Transversal cross-section of an adult sporophyte showing the meristoderm (me), cortical cells (cc), and medulla (md); **b** fresh released zoospores from sporangia (sp) from a mature sporophyte; **c** transmission electron microphotograph of a gametophyte cell showing nucleus (n), nucleolus (nu), chloroplasts (ch), cell wall (cw), golgi bodi (g), mitochondria (m), and physodes (p); **d** female gametophyte culture; **e** male gametophyte culture; **f** early-stage embryonic sporophytes (indicated with triangles) still attached to female gametophyte (gf). Scale bar: **a** 50 µm, **b** 5 µm, **c** 2 µm, **d**–**e** 10 µm, and **f** 20 µm

**Fig. 3:**
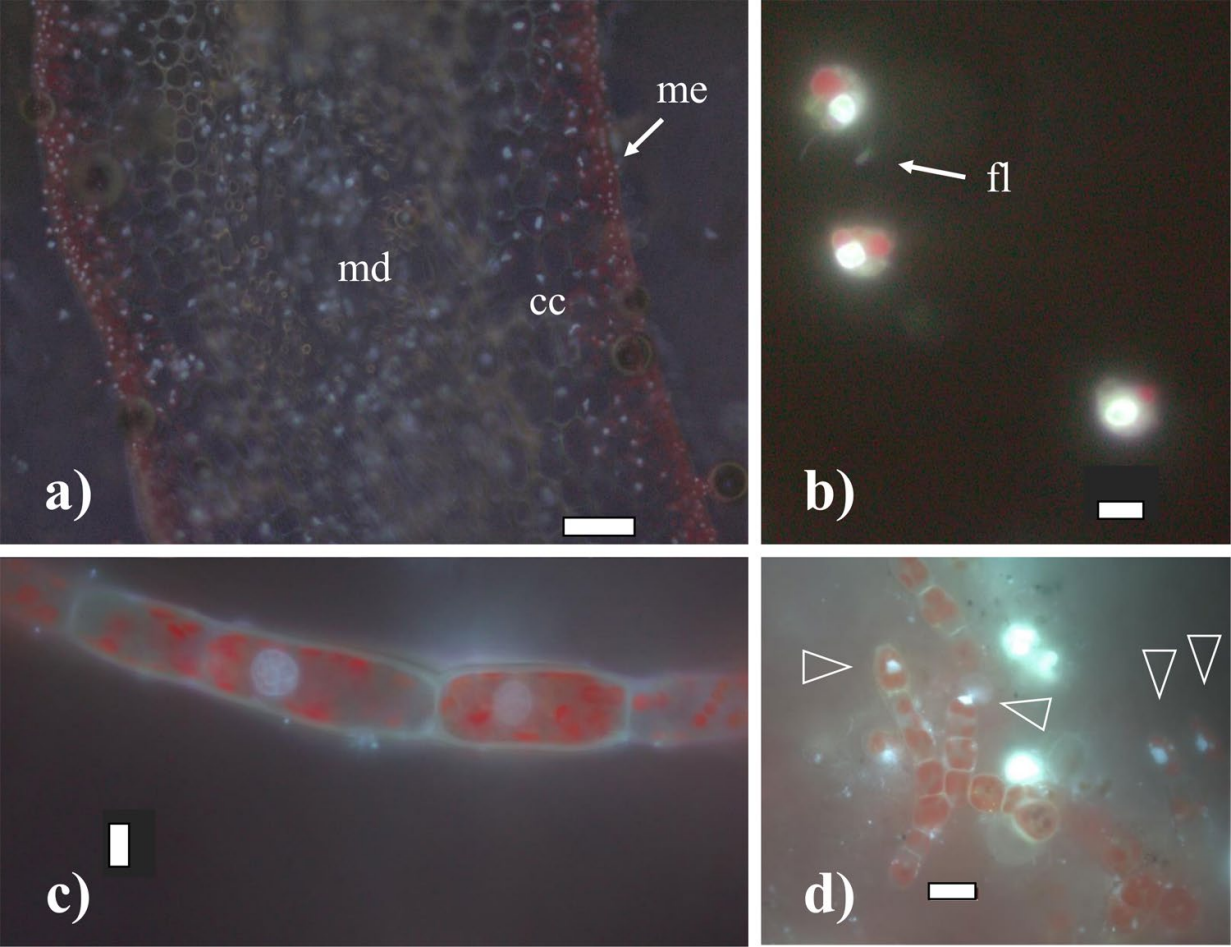
Cells of *Saccharina latissima* under fluorescence, stained with DAPI without methacarn treatment showing chloroplasts in dark red and stained nuclei in light white/blue: **a** transversal cross-section of an adult sporophyte showing the meristoderm (arrow: me), cortical cells (cc) and medulla (md); **b** released zoospores with chloroplasts, a nuclei and flagella (arrow: fl); **c** female gametophyte filament with cylindrical cells chloroplasts; **d** male gametophyte with rounded cells and many chloroplasts, nuclei indicated with triangles. Scale bar: **a** 100 µm, **b**–**c** 5 µm, and **d** 10 µm

**Fig. 4:**
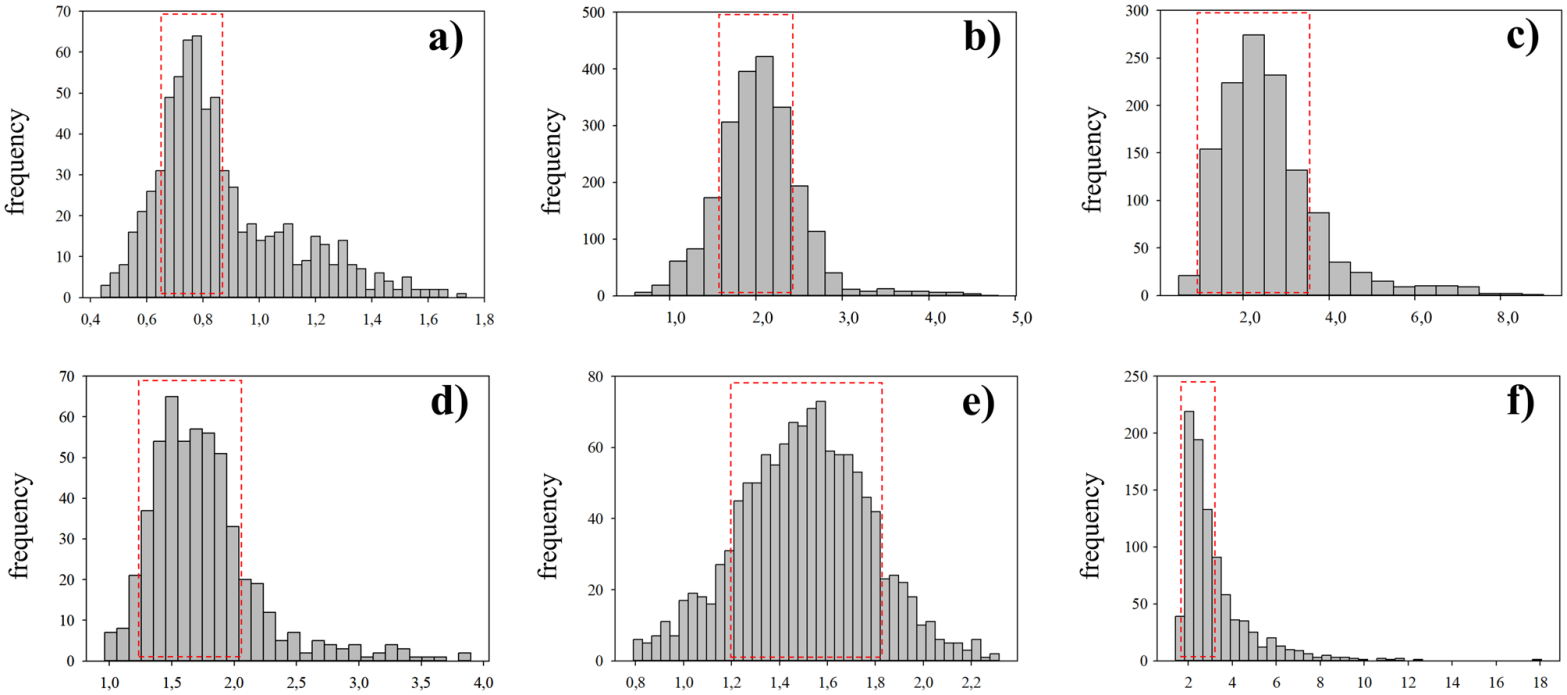
Frequency distribution of nuclear DNA contents in different life cycle stages of *Saccharina latissima*, as measured by DAPI staining and fluorescence microscopy and image analysis, including haploid zoospores (**a**), haploid male gametophytes (**b**), haploid female gametophytes (**c**), diploid early-stage sporophytes (**d**), and older sporophytes, divided in meristoderm (**e**) and cortex/medulla cells (**f**). For each cell type *n* > 100. For calculation of mean values of the nuclear DNA content, the peak frequency class was identified, and the neighboring frequency classes which presented frequencies above the 50% of that of the peak were included (red squares)

**Fig. 5:**
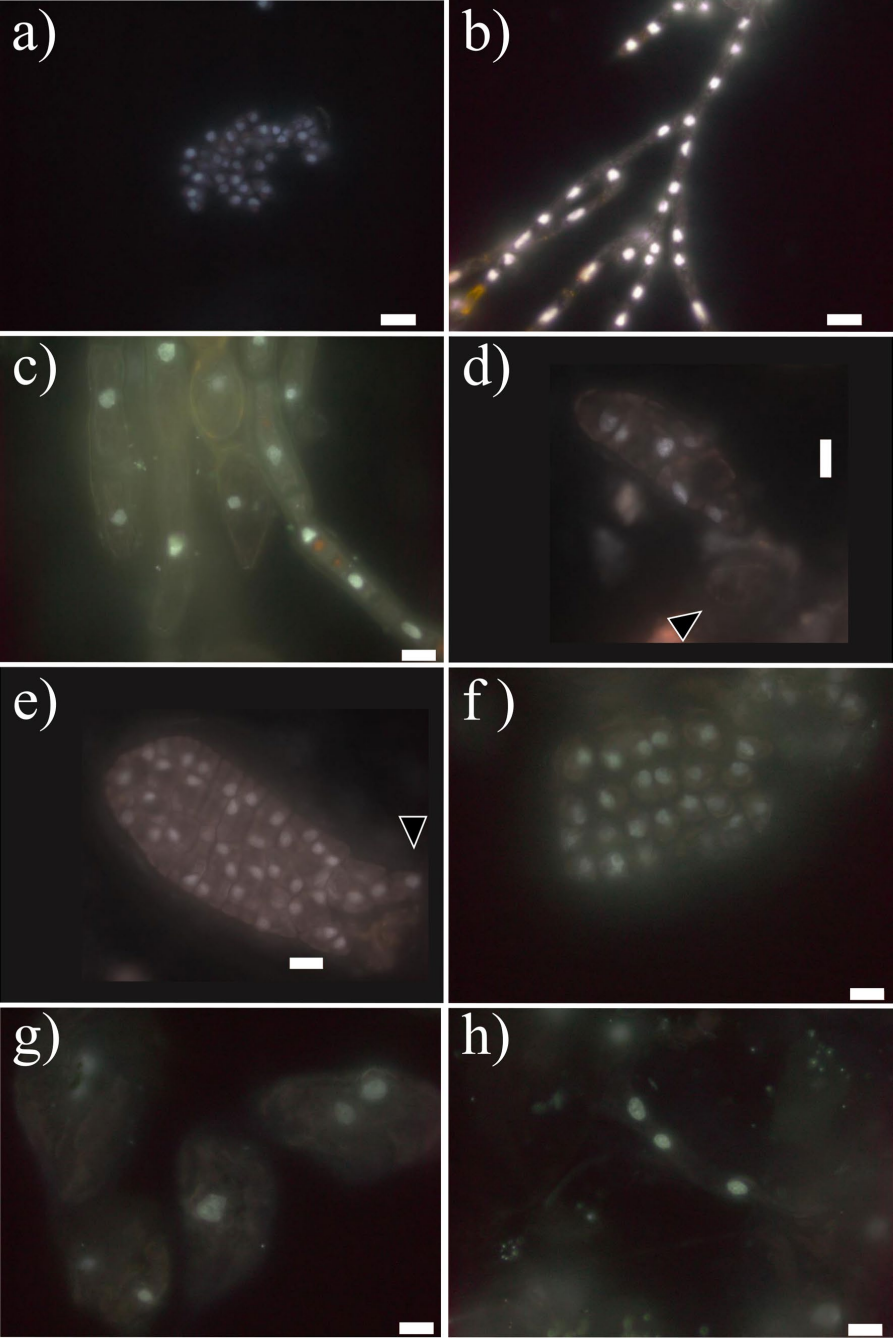
Different DAPI-stained nuclei after methacarn treatment: **a** from released zoospores; **b** from male gametophytes; **c** different forms and sizes from female gametophytes; **d**–**e** from early-stage development of embryonic sporophytes including a few celled sporophyte still attached to the female gametophyte (black arrow, **d**) and a further developed sporophyte with rhizoidal cells (black triangle, **e**); and **f**–**h** from different tissue cells of full developed sporophyte including meristoderm (**f**) and large cortical (**g**) and long medullar cells (**h**). The scale bar in all pictures corresponds to10 µm

In terms of morphology, zoospores were small (around 5 µm), round, biflagellate, and heterokont and contained 2–3 chloroplasts as described previously (Henry and Cole 1982a; see Figs. 2b and 3b). Zoospore cells were very regular in size and form, as were their nuclei, although a degree of variation in nucleus forms was observed (Figs. 4a and 5a). The final frequency distribution of nuclear DNA levels of the total sample (3 individuals together) used for the determination of the C-value is shown in Fig. 4a. Our results reveal that the C-value of released zoospores of *S. latissima* from Frøya was 0.76 ± 0.07 pg (*n* = 387, Table 2).

**Table 2:** Measurements of nuclear DNA content in picograms (pg, mean ± standard deviation of all nuclei) with corresponding C-value for different life phase stages of the brown macroalga *Saccharina latissima*, obtained by comparison of fluorescence intensity values with those of chicken erythrocytes. In case of zoospores (released and unreleased), *n* is the number of parental sources; in the case of gametophytes, it is the number of single-gametophyte cultures, and in the case of sporophytes, it is the number of individuals. If we detected cell with higher C-value, although in no large numbers, we expressed it with “ + ” or “ +  + ” if more abundant. The average of relative nucleus size (NRS) obtain in this study is given (± standard deviation)

Life phase	*n*	n° nuclei	NRS	1C	2C	4C	8–16C
Zoospores (released)	3	387	0.044 ± 0.01	0.76 ± 0.07	+	-	-
Zoospores (unreleased)	1	64	-	0.79 ± 0.05	-	-	-
Male gametophytes	6	1464	0.043 ± 0.01	-	2.01 ± 0.22	+	-
Female gametophytes	5	946	0.084 ± 0.03	-	2.27 ± 0.56	+ +	+
Sporophytes: embryonic	25	408	0.095 ± 0.02	-	1.64 ± 0.21	+	-
Sporophytes: meristoderm	7	905	0.129 ± 0.07	-	1.51 ± 0.16	-	-
Sporophytes: cortex/medulla	7	629	-	-	2.40 ± 0.41	+ + +	+ + +

**Fig. 6:**
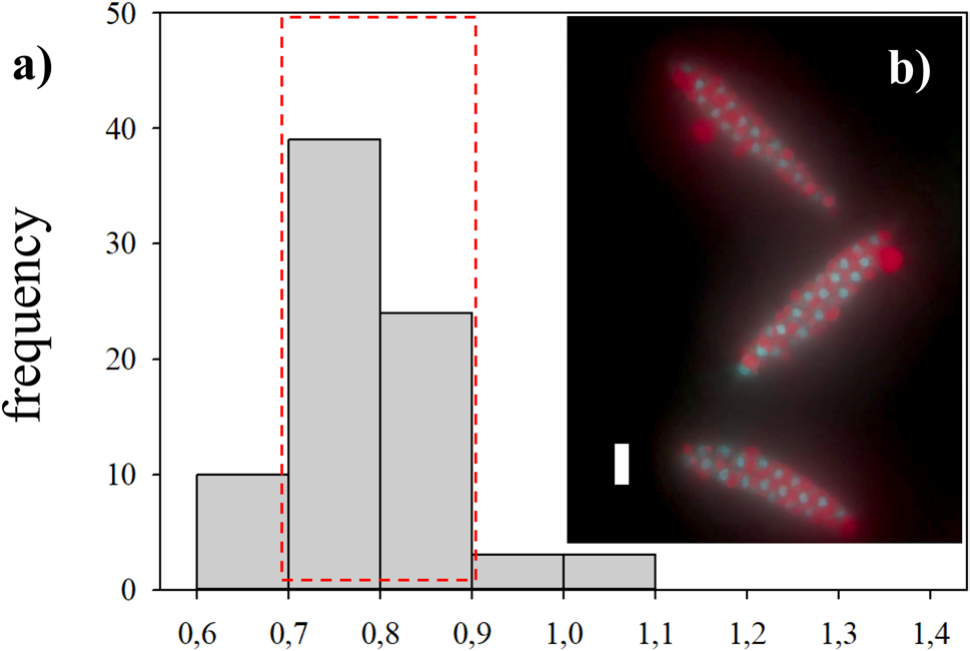
**a** Frequency distribution of nuclear DNA content in zoospores disaggregated from unreleased sporangia of *Saccharina latissima*, measured and calculated as stated before. Inlay on the right: **b** DAPI-stained nuclei after methacarn treatment from unreleased zoospores in three sporangia is depicted. Zoospores are surrounded by a liquid red substance that interferes with the fluorescence signal. The scale bar corresponds to 10 µm

The presence of a fertile sporophyte at sampling allowed us to determine the nuclear DNA content in unreleased zoospores from sporangia. Zoospores disaggregated from unreleased sporangia presented a C-value of 1C, which matched the previous result of released zoospores (0.79 ± 0.05 pg; *n* = 64, Fig. 6).

Gametophytes had uniseriate filaments of spherical to cylindrical cells with several chloroplasts (Fig. 2c). Filaments were ramified and varied in size (Fig. 2d, e). Male gametophytes presented very compacted cells and/or narrow and “straight” filaments. Their cells and respective nuclei were more or the less regular in shape, bigger in size than zoospores, although in some cases, nuclei appeared to be of similar size as those of zoospores (Fig. 5a, b); nevertheless, their C-value was always larger than the latter (Fig. 4a, b). The nuclear DNA content estimate for six male gametophyte cultures was 2.01 ± 0.22 pg, which is 2.6 times as much as that observed in zoospores, with a resulting peak corresponding to 2C (*n* = 1464, Table 2, Fig. 4b). Female gametophytes showed a larger variation in form and size than cells of male gametophytes, with some nuclei presenting small spherical forms, while others were large, elongated, and asymmetric (Figs. 3c and 5c). Female gametophyte cultures presented a nuclear DNA content of 2.27 ± 0.56 pg, which is 3.0 times as much as in zoospores with a peak corresponding to 2C (*n* = 946, Table 2, Fig. 4c). Larger oval nuclei with higher pg values (up to 8.7 pg) were observed in large gametophyte cells, although they were less common.

By crossing of female and male gametophytes, we obtained several embryonic early-stage sporophytes which were analyzed (Fig. 2f). Some of the sporophytes were made up of very few cells (2, 4, 6, etc.), and some were still associated with the female gametophyte (Fig. 5d). Others already started to develop a microscopic frond, had a monostromatic micro-lamina with several cells (more than 100), and had rhizoidal cells at the base (Fig. 5e). Only one sample was larger than these, reaching between 1 and 2 mm, already differentiated in a microscopic stipe and frond. Embryonic sporophytes presented small, compacted, and pigmented cells (Figs. 2f and 5d, e) and presented a nuclear DNA content of 1.64 ± 0.21 pg, which is 2.2 times as much as in zoospores (*n* = 408, Table 2). Very few nuclei were 4C, probably under division (Fig. 4d). In very young sporophytes with few cells, nuclei were slightly irregular in shape (Fig. 5d), but once a frond was established, meristoderm nuclei turned more regular (Fig. 5e).

Large and older mature sporophytes presented a large variation in cells sizes, with more regular and smaller cubic, compacted and heavily pigmented cells at the meristoderm, large and irregular, large spherical to ovoid cortical cells, and long “filaments” belonging to the medullar tissue (Figs. 2a and 3a). Nuclei varied in sizes accordingly (Fig. 5f–h). The DNA content of the nuclei in the meristoderm corresponded mostly to 2C (1.51 ± 0.16 pg, *n* = 905), while the large spherical to oval and elongated nuclei in the cortical and medullar tissue had higher DNA content, corresponding to 2-16C (Figs. 5f–h and 6). No obvious geographical (ecoregion) differences were observed among the sporophytes C-value (data not shown).

**Fig. 7:**
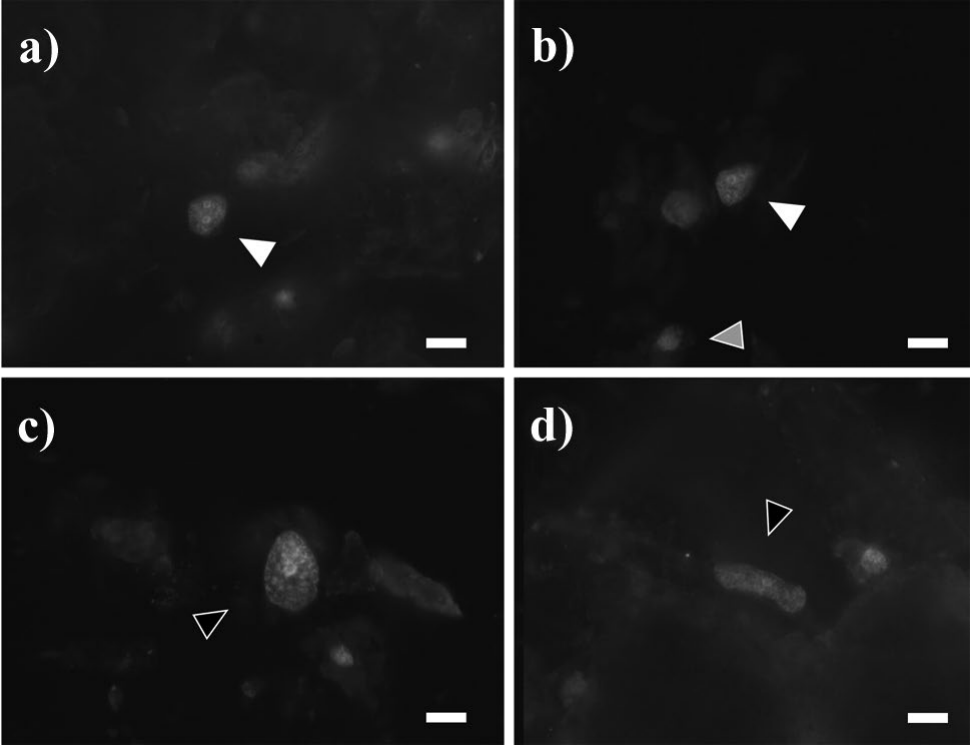
Large nuclei of cortical and medullar cells of sporophytes of *Saccharina latissima*, treated with methacarn and stained with DAPI. **a**–**b**) detail of dividing nuclei, which corresponded to 4C (white arrow) in comparison with a 2C nuclei (gray arrow). **c**–**d** extra-large nuclei with no signs of division at the cortex (**c**) or medulla (**d**), which presented 8C. Scale bar 10 µm

In all the samples investigated, most of the cells observed in the studied life cycle stages appeared to be uninucleate, even though some presented extremely high C-value as 16C (Fig. 7c and d). Nevertheless, some nuclei appeared to be in division as well (Fig. 7a, b).

**Fig. 8:**
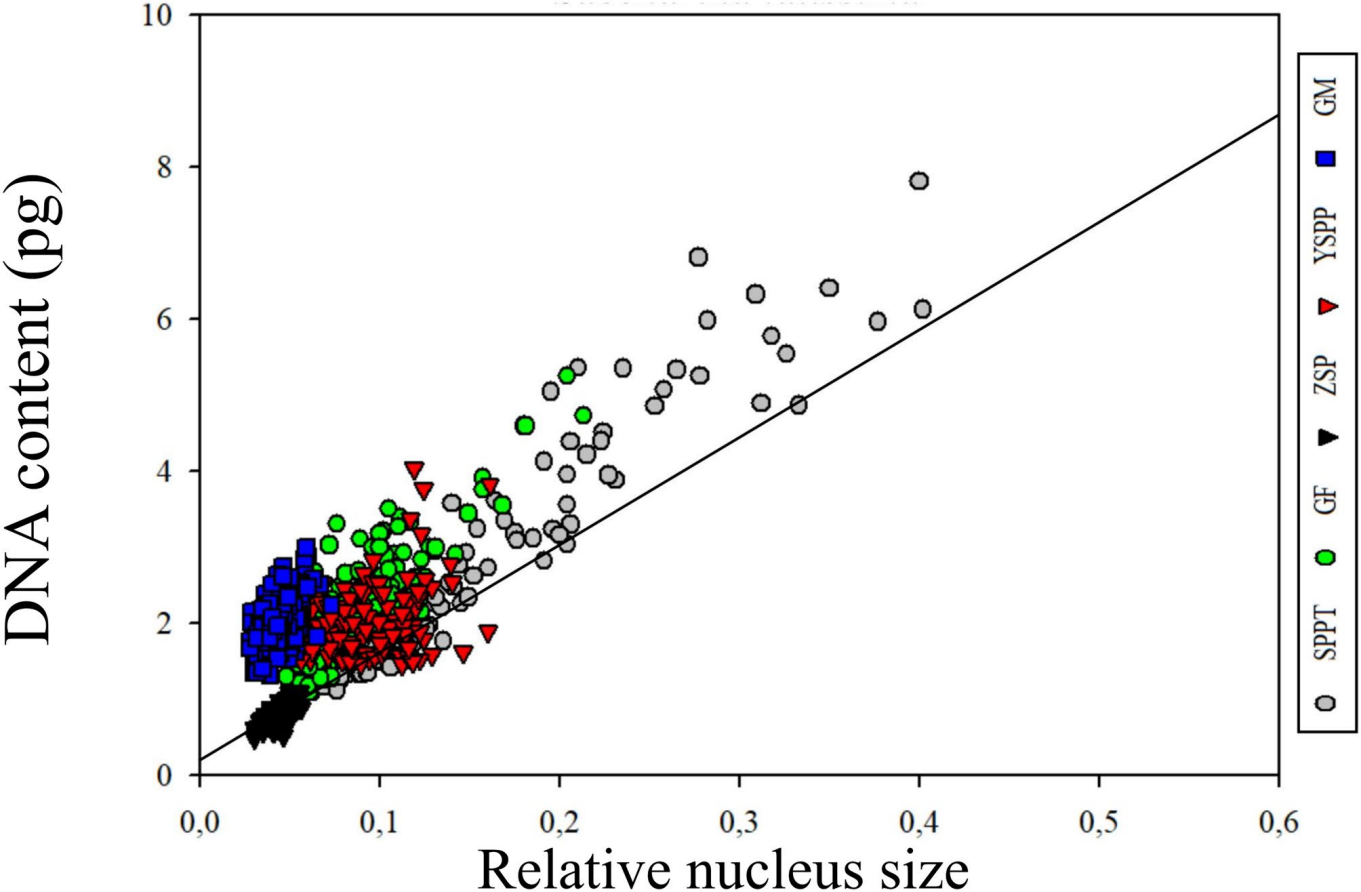
Relative nucleus size vs DNA content of cells at different life stages of *Saccharina latissima*. There is a positive correlation between nuclei size and nuclear DNA content. The smaller nuclei corresponded to zoospores (ZSP) and male gametophytes (GM), followed by female gametophyte (GF) and early-stage sporophytes (YSPP). The larger nuclei corresponded to older sporophyte tissue (SPPT). Nevertheless, male gametophytes presented almost three times the amount of DNA in comparison with zoospores. A regression line is showed for zoospores

Image analysis allowed us to measure a relative nucleus size of the studied cells and correlate them with the DNA content obtained for each nucleus at different life stages of *S. latissima*. The smaller nuclei corresponded to zoospores and male gametophytes, followed by female gametophyte and early-stage sporophytes. The larger nuclei corresponded to large cells in older sporophyte tissue (Fig. 8). We observed a positive correlation between nucleus size and DNA content in samples of the different life stages, although the correlation is not linear if we consider the nuclear DNA content of the small-sized zoospores and small-sized male gametophyte cells.

## Discussion

Despite the ecological, economic, and evolutionary importance of kelps, we currently have a very limited knowledge of their genetics and metabolism (e.g., iodine metabolism or alginate-producing pathway) (Ye et al. 2015; Müller et al. 2016). So far, the C-value of only a few species of the order Laminariales have been reported (Table 1), but to our knowledge, there is no systematic comparison of the nuclear DNA content of a Laminarialean macroalgae through their life cycle. In practice, most published DNA content values are for 2C diploid nuclei and most 1C and 4C values are extrapolated (Kapraun 2005). This is the first study to determine C-value across the life cycle of a Laminarian kelp (with the only exception of gametes) and relate it to its nucleus size.

### C-value and Nuclear Size in Zoospores and Gametophytes

Our observations on anatomy and internal structure of the different life stages of *S. latissima* growing at Frøya (Mid-Norway) agree with previous anatomical studies for the genus (see Sykes 1908; Drew 1910; Killian 1911; Naylor 1956; and Chung et al. 1987).

Members of Laminariales are characterized by a heteromorphic diplohaplontic life cycle (Fig. 1) where both independent haploid and diploid phases exhibit vegetative growth (Oppliger et al. 2014). In theory, nuclei of diploid somatic sporophyte cells have a DNA content of 2C in the G1-phase and 4C in the G2-phase, as observed in the Fucalean *Fucus* (Sjøtun et al. 2017). We observed the lowest DNA content in zoospores (0.76 ± 0.07 pg), a similar value to that found in previous studies of species in the genus (1C, Table 1). On the contrary, cells of *S. latissima* male and female gametophyte cultures showed 2.2 and 3.0 times higher DNA content, respectively (Table 2).

Several sexually dimorphic traits have been described previously in brown algae (Bolton 2010; Luthringer et al. 2014). Morphological differences in cell and nucleus size between sexes of gametophytes were already shown by Kanda (1936), Naylor (1956), and Evans (1965), who studied different Laminariales species (including *S. latissima*). The authors reported that with an average diameter of 4.5–5 µm, the nuclei of female gametophytes are larger than those of the male gametophytes, which have an average diameter of 3.5 µm, but smaller than those of sporophyte vegetative cells, which are 7.5–8 µm. The relative nucleus size obtained in our study confirmed this difference (0.043 ± 0.01 in male gametophytes against 0.084 ± 0.03 in female gametophytes, Table 2). Furthermore, Bi and Zhou (2014) observed in *S. japonica* that the chromosome size of female gametophytes is larger than that of male gametophytes (around 0.78–2.61 µm and 0.38–1.57 µm, respectively). Later, Müller et al. (2016) suggested the presence of sex-specific polyteny in *M. pyrifera* gametophyte cultures, in which female gametophytes had approximately the double nuclear DNA content in comparison with the male gametophytes. The fact that we observe a DNA content in gametophytes that is more than twice as high as in zoospores, and differ between males and females, suggests that polyteny is also present in gametophytes of *S. latissima*.

### C-value and Nuclear Size in Sporophyte Cells

**Fig. 9:**
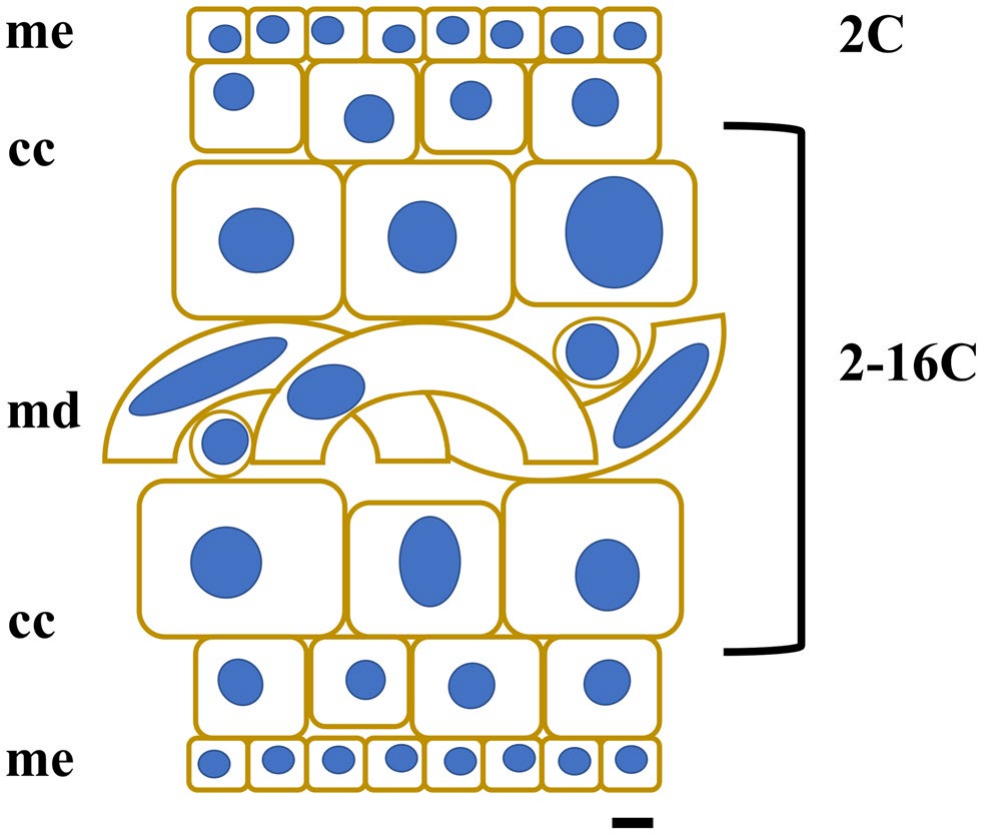
Diagram of a transversal cut of a sporophyte of *Saccharina latissima*, showing different cell forms and sizes. Meristoderm with small, compact cuboid cells (me), followed by larger cortical cells (cc) with increasing and variable sizes, and an internal medullar section, with large, entangled cells (md). The C-value obtained in the present study for the different tissue cells and a schematic illustration of nucleus sizes is also shown (scale bar is 10 µm)

Young sporophytes and the meristoderm tissue of older sporophytes had a DNA content corresponding very well to twice the content in zoospore nuclei. It also corresponds well with 2C in Laminariales (Table 1), while in older sporophytes, some tissues (cortex/medulla) had cells with DNA content ranging from 2C and up to an estimated 16C. Internal differences in cell size across the sporophyte tissue were already showed by Davies et al. (1973) and Chung et al. (1987), and Kapraun (2005) mentioned the presence of large nuclei in older medullary cells of *S. latissima*, which he was not able to quantify. The large variation in DNA content observed in older sporophyte tissue (of European plants) — of at least four ploidy levels (Table 2, Fig. 4d and f) — confirmed the observations made by Garbary and Clarke (2002) on Nova Scotian *Alaria* and *Saccharina* in terms of different internal C-value in sporophyte tissue. The authors suggested that given the large number of 8C and 16C nuclei in *S. latissima*, their absence in previous studies with European plants was anomalous (see Ar Gall et al. 1996). In our study, Norwegian *S. latissima* presented a uniform 2C value in the meristoderm (contrary to the 2-8C from the Nova Scotian individuals). Larger cortical and medullar cells presented higher C-values of 4C, 8C and 16C (Fig. 9).

### Correlation Between Nuclear Size and DNA Content

Our results show that the total DNA content of the cell is not only a function of its generation, but of cell and nuclear size, as also stated by Goff and Colemann (1990), and later observed in small cortical cells of the red macroalgae *Gelidium chilense* (Montagne) Santelices and Montalva (Salvador-Soler et al. 2016). Effectively, we observed a positive correlation between nucleus size and DNA content at samples of the different life stages (Fig. 8). The smaller size of nuclei corresponded to zoospores and male gametophytes, followed by female gametophyte, early-stage sporophytes, and finally, the older sporophyte tissue with the larger nuclei (and the larger nuclear DNA content) (Table 2). Nevertheless, even though nucleus size of male gametophytes were apparently as small as in zoospores, they presented more than double the amount of DNA in comparison with them (Fig. 8). We do not know why males can escape this positive relationship between nuclear size and the amount of DNA. Proposed mechanistic theories for existence of this type of relationship are varied and invoke a variety of linking factors, ranging from simple space constraints to potential osmolyte function of nucleotides and optimal transport of RNA from nucleus to the cell (Connolly et al. 2008).

Intracellular structure may also determine the nuclear DNA content and, as suggested by Salvador-Soler et al. (2019), may produce the wide ranges of DNA content observed in other Laminariales (i.e., cortical, or medullar cells of *Alaria esculenta*, *Lessonia spicata*, or *Macrocystis pyrifera*). Garbary and Clarke (2002) suggested, based on the ultrastructure studies of Davies et al. (1973) and Chung et al. (1987), that cortical cells of *S. latissima* have similar DNA content as meristoderm cells because cytoplasmic volumes are similar; the difference in cell size is due to differences in the volume of the vacuoles. The authors showed a gradient of increasing vacuolation in meristoderm, medullary, and cortical cells, with the latter having especially prominent vacuoles. This may be true for some of the *S. latissima* cells. From our observations under transmission electron microscopy, we observed that vacuoles were not particularly notorious in our gametophytes (Fig. 2c), which could explain larger nuclei in larger cells (male vs female gametophyte cells).

### Possible Functions of Higher DNA Content

Examples of higher DNA content and/or somatic polyploidy are particularly prevalent in plants with small genome sizes, raising the possibility that high ploidy may be needed for certain aspects of plant growth or function, which include a requirement for increased cell size to achieve a particular morphology, mass, metabolic output, or the use of increased gene copy number to cope with environmental damage (reviewed by Edgar and Orr-Weaver 2001). Somatic polyploidy (endopolyploidy) is a common response to UV stress in natural plant populations (Zedek et al. 2020). In this sense, somatic polyploidy could be very relevant for kelp ecology: 1) Fast growth of annual kelp sporophytes is critical for escaping herbivory (Poore et al. 2014), and 2) kelp meiospores and gametophytes — critical for the maintenance of the population after annual sporophytes decay — are particularly vulnerable to fluctuations in environmental conditions (Xu et al. 2015; Augyte et al. 2019). It would be interesting, for example, to evaluate the environmental effect on the C-value in future scenarios under acidification or global warming. Scenarios that could affect plant size, growth, and the ultrastructure of macroalgae cells, as for example, the vacuolar volume (i.e., Xu et al. 2015; Augyte et al. 2019).

As mentioned before, the characterization of nuclear DNA content can give us key information for a better understanding of the life history in kelp species related to certain environmental or biotic conditions, and further on, it could be linked to patterns of evolution (Klimova and Klochkova 2021). In this sense, polyploidy is often associated with asexuality, and asexuality can be related to stress (Oppliger et al. 2014). In kelps, it has been observed that several species can produce sporophytes through parthenogenesis, directly from non-fertilized female gametes (Nakahara and Nakamura 1973; Lewis et al. 1993; Ar Gall et al. 1996; Oppliger et al. 2007, 2014; Müller et al. 2019), and that changes in the ploidy of those parthenosporophytes could be generated through different cellular processes like apomeiosis, automixis, or endomitosis (endoreduplication) at specific stages of the development (Goecke et al. 2020, and references therein). Interestingly, differences in gamete size may be one of the factors that determine whether a gamete can undergo asexual reproduction through parthenogenesis, should it fail to encounter a gamete of the opposite sex (Heesch et al. 2021). We do not know if there is a specific role regarding this and the observed increase in nuclear DNA content of female gametophytes.

On one hand, the occurrence of meiosis of apomictic life histories can be established unequivocally with fluorescence microscopy and image analysis, and the exact site of meiosis readily ascertained (Goff and Coleman 1990). On the other hand, in view of experiments of sexual or parasexual hybridization among close but significantly different genomes (Goecke et al. 2020, and references therein), fluorescence microscopy and image analysis may help to identify putative hybrids (Le Gall et al. 1993). As an example, Klochkova et al. (2017) demonstrated the first evidence of parthenogenic sporophytes of the Laminarialean kelp, *Tauya basicrassa* N.G. Klochkova & T.N. Krupnova, occurring naturally in form of a dwarf (haploid) thallus in the Sea of Okhotsk (Russia).

Fluorescence microscopy and image analysis is time consuming, but it allowed us also to examine directly and in detail into the macroalgae tissue. A feature that can be a clear advantage to localize endoreduplication, in comparison with other techniques used for inferring genome size or ploidy level, i.e., chromosome counting or flow cytometry (Katagiri et al. 2016). It may require less biomass as well, although fewer nuclei can be characterized compared to flow cytometry. Chromosome counting does not allow to measure the differences in DNA amount observed in female and male gametophytes vs zoospores as in the present study. Nevertheless, and besides difficulties of this technique using macroalgae (Goff and Coleman 1990; Deshmukhe and Tatewaki 1993), it is a crucial technique to determine the final ploidy stage of cells.

## Conclusion

Ploidy variants are useful in aquaculture and crop breeding, as it has turned to be a tool to increase yield, introduce sterility, and modify specific traits with a large economic impact. If we want to achieve an increase in yield through genetic improvement, but at the same time, take care of the local genetic heritage by preventing hybridization with wild populations, it will be useful to obtain a detailed understanding of and be able to control the regulation of the cell cycle and sporophyte development in Laminariales. This is the first C-value study which covered the whole life cycle of a Laminarian kelp, with the only exception of gametes. The observed variation of DNA content values may be further utilized in creating ploidy variants for future environmentally friendly kelp mariculture.
